# The Safety and Effectiveness of Single-Stage, Vessel-Preserving, Laparoscopic Orchiopexy for Intra-Abdominal Testes in Pediatric Patients: A 10-Year Single-Center Experience

**DOI:** 10.3390/jcm13072045

**Published:** 2024-04-01

**Authors:** Zenon Pogorelić, Josipa Šitum, Tomislav Barić, Marijan Šitum

**Affiliations:** 1 Department of Surgery, School of Medicine, University of Split, 21000 Split, Croatia; 2 Department of Pediatric Surgery, University Hospital of Split, 21000 Split, Croatia; 3 Department of Urology, University Hospital of Split, 21000 Split, Croatia

**Keywords:** cryptorchidism, laparoscopic orchiopexy, intra-abdominal testis, vessels preserving orchiopexy, single-stage orchiopexy, children

## Abstract

**Objectives:** Intra-abdominal testes are located in a variety of intra-abdominal positions, most less than 2 cm from the internal ring. Various surgical techniques of laparoscopic orchiopexy have been described to date. The aim of this study was to evaluate the safety and long-term efficacy of a single-stage, vessel-preserving, laparoscopic orchiopexy for intra-abdominal testes in pediatric patients. **Methods:** A retrospective search of the medical records of 32 children (34 testes) who underwent single-stage, vessel preserving, laparoscopic orchiopexy for intra-abdominal testes between 1 January 2014 and 31 December 2023 was performed. Single-stage laparoscopic orchiopexies were performed in all patients for whom sufficient length of the spermatic cord was achieved during the procedure. The volume of each patient’s testes was measured using ultrasound before and 6 months after laparoscopic orchiopexy. The main outcome of this study was testicular volume before and after the procedure. The secondary outcomes were the occurrence of early and late complications, the duration of surgery, and the length of hospital stay. **Results:** The median age at the time of surgery was 10 months (interquartile range—IQR 9, 13). The majority of the children (*n* = 24; 75%) were less than 12 months old at the time of surgery. A normal testis was found in 24 patients (70.6%), while a hypotrophic testis was visible in 10 cases (29.4%). The majority of the testes were located near the internal ring (*n* = 19; 55.9%), while in the remaining cases, the testes were located near the iliac blood vessels. The median duration of the surgical procedure was 37.5 min (IQR 33, 42.5). The duration of hospitalization was one day for all the children. No intraoperative complications were observed. One child had a wound infection at the site of the umbilical trocar, which was treated conservatively. In two cases (5.5%), testicular atrophy was detected during long-term follow-up. In three cases, the testis was found in a higher position in the scrotum during the follow-up period, but in two cases, the position was normal during the follow-up period, while in one case, the position in the scrotum remained unchanged. At long-term follow-up with a median of 35 months (IQR 19, 60.5), the overall success rate was 94.5%. The median testicular volume at 6-month follow-up increased from 0.31 mL (IQR 0.28, 0.43) to 0.40 mL (IQR 0.33, 0.53) (*p* = 0.017). **Conclusions:** Single-stage, vessel-preserving, laparoscopic orchiopexies for intra-abdominal testes are safe and effective in pediatric patients in whom adequate spermatic cord length can be achieved during the procedure.

## 1. Introduction

Cryptorchidism is a congenital condition in which the testicles do not descend into the scrotum. In about 80% of patients with cryptorchidisms, the testicle is palpable (inguinal retention), while in the remaining 20% of cases, the testicle is non-palpable (intra-abdominal retention or testicular agenesis) [1]. At the time of birth, the incidence of undescended testicles in boys is 4–5%, while the incidence of undescended testicles drops to 1–2% after the third month of life [1]. The reason for this is the spontaneous descent of the testicles into the scrotum in about half of the cases of boys with undescended testicles due to the hormonal effect. The most important consequences of cryptorchidism are infertility and the occurrence of malignant testicular tumors [1,2]. In addition, several studies have shown that testicular torsion and trauma are more common in patients with an undescended testes [3,4].

An undescended testicle is not palpable in the scrotum, but it usually is in the groin. In addition, a hypoplastic and less-developed scrotum is often visible on the ipsilateral side of the retained testicle. If the testicle cannot be palpated, an ultrasound examination of the scrotum is performed. If the ultrasound examination shows the same, no further diagnostic work-up is required. If the testicle cannot be palpated or visualized via an ultrasound examination, an MRI of the abdomen and groin and/or a laparoscopic exploration can be performed. Laparoscopic exploration is the gold standard for determining the location of the testicle in the case of intra-abdominal retention [5].

There are conservative, hormonal, and surgical treatments for an undescended testicle. Conservative treatment involves observing a child with undescended testicles expectantly for the first six months, as in around half of cases, spontaneous testicular descent occurs under the influence of a temporarily elevated testosterone level (usually in the first 3 months of life). Hormonal treatment involves the use of gonadotropins and luteinizing hormone-releasing hormone (LHRH), which increase testosterone levels and other testicular hormones, leading to testicular descent. This type of approach and treatment has proven to be controversial in the literature. It has been concluded that this type of treatment can only be successful in retractile or acquired ascending testes, while it is usually unsuccessful in congenital cryptorchidism [5,6].

The ideal time for an orchidopexy has changed over the years. The literature shows that orchidopexy up to the age of 10 or 11 years greatly reduces the risk of testicular tumors [7]. Orchidopexy is not recommended before the first six months of life, as in about half of the cases, there is a possibility of spontaneous descent of the testicles into the scrotum. According to a systematic review of the literature, the ideal time for orchidopexy is the 6th to 12th month of life if there is no irreversible damage to the testicular germinal epithelium and spermatogenesis [7].

The choice of surgical treatment depends primarily on the type of cryptorchidism (inguinal or intra-abdominal). Thus, in most international centers, when dealing with the surgical treatment of cryptorchidism, the prevailing opinion is still that, in the case of inguinal retention, a classic or open orchidopexy should be performed [5]. In the more recent literature, laparoscopic orchidopexies are mentioned as a possible alternative to open orchidopexies [8,9,10]. The results after laparoscopic orchidopexies demonstrate a lower risk of complications such as testicular atrophy and wound infection and greater success in maintaining the position of the testicle in the middle or lower part of the scrotum, without subsequent retraction [10].

In the case of intra-abdominal retention of the testis, either unilateral or bilateral, the gold standard is laparoscopic exploration of the abdominal cavity, identification of the position of the testis, and finally laparoscopic orchidopexy [11]. A laparoscopic orchidopexy can be performed in one or two stages, depending on surgeon’s preferences and the position of the testicle. In a single-stage laparoscopic orchidopexy, the vas deferens and the spermatic blood vessels are detached and mobilized, and the testicle is then lowered into the subcutaneous pocket of the scrotum in the same act. The prerequisite for a single-stage laparoscopic orchidopexy is that the testicle can be pulled to the contralateral inguinal opening without tension after mobilizing the vas deferens and spermatic vessels [12,13].

In the case that the testicle after mobilization of the blood vessels and the vas deferens cannot be pulled to the contralateral inguinal opening, a two-step laparoscopic orchidopexy is performed in most centers. One of the most commonly performed two-step laparoscopic orchidopexies in the past was the Fowler–Stephens technique, in which the spermatic blood vessels are resected laparoscopically in the first stage, followed after six months by the second stage of lowering the testicle into the scrotum. Sometimes, if sufficient length and mobilization of the testicle is achieved, it can also be lowered in the first stage. In addition, this method can also be performed via the classic open approach [14,15].

Recently, Shehata’s laparoscopic stepwise traction orchidopexy gained popularity among pediatric surgeons. In the first stage, after mobilization of the vas deferens and spermatic vessels, the testicle is temporarily fixed retroperitoneally without tension with a suture that is slightly higher and medial to the anterior superior iliac spine. The second stage of this method is performed usually 12 weeks later and is based on the descent of the testicles into the scrotum. Although this technique may be used for all cases of intra-abdominal testes, it is most commonly used in cases of high intra-abdominal testicular retention, where the testicle lies below the lower edge of the kidney [15]. In addition, a recent meta-analysis showed that the Shehata technique demonstrated better performance than staged Fowler–Stephens orchiopexies regarding the overall success and atrophy rates [16].

The aim of this study was to evaluate the safety and long-term efficacy of single-stage, vessel-preserving, laparoscopic orchiopexies for intra-abdominal testes in pediatric patients.

## 2. Methodology

### 2.1. Patients

**Figure 1:**
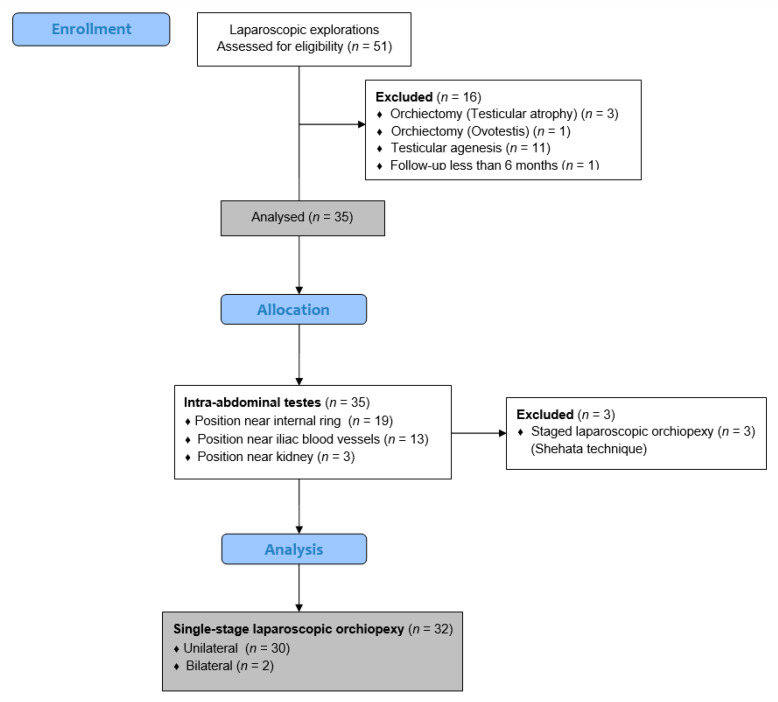
Flowchart of this study.

A retrospective search of medical records for children who underwent laparoscopies for undescended intra-abdominal testis between 1 January 2014 and 31 December 2023 at the Department of Pediatric Surgery, University Hospital of Split, was performed. A total of 51 patients were identified, but 19 children were excluded from further analysis because they met one or more of the exclusion criteria. Finally, 32 children (34 testes) met the inclusion criteria and were included in this study. The inclusion criteria were pediatric patients undergoing single-stage laparoscopic orchiopexies for unilateral or bilateral an intra-abdominal cryptorchid testes. The patients who underwent a staged laparoscopic traction-orchiopexy, the patients who underwent laparoscopic orchiectomy/spermatic cord excision for testicular atrophy/agenesis, the patients who were converted to an open procedure, the patients with a follow-up period of less than six months, and the patients with incomplete data in their medical records were excluded from this study. The flow diagram of this study is shown in Figure 1.

### 2.2. Ethical Aspects

This study was conducted in accordance with the Declaration of Helsinki of the World Medical Association, and the Institutional Review Board of our hospital approved the study (approval number: 500-03/23-01/207; date of approval: 26 October 2023).

### 2.3. Outcomes of the Study

The main outcome of this study was testicular volume before and after the procedure. The secondary outcomes were the occurrence of early and late complications, the duration of surgery, and the length of hospital stay (LOS). The frequency of unplanned return to the operating room (uROR) [17] and the number of readmissions within 30 days of index surgery (ReAd) [18] were used as quality-of-care indicators in our department.

### 2.4. Study Design

According to the current literature and the guidelines of our department, the surgery was planned at the age of 6 to 12 months [7]. Only the patients who appeared for the first examination after the age of 12 months were operated on later than the indicated interval. Magnetic resonance (MR) scans were performed on each patient prior to surgery to determine the location of the testes. Prior to the surgery, an abdominal ultrasound (US) was performed to determinate the testicular volume. A single-stage laparoscopic orchiopexy was performed in all patients in whom sufficient length of the spermatic cord was achieved during the procedure. The patients for whom the spermatic cord was too short due to the high position of the testis underwent staged laparoscopic traction orchiopexies (Shehata technique) [15] and were excluded from further analysis. For each patient included in this study, the following data were collected: age, weight, height, body mass index (BMI), lateralization of the undescended testis, concomitant diseases, location (above the internal ring, next to the iliac blood vessels, or next to the kidney), size (normal, hypotrophic, or atrophic) of the testis, presence of early (bleeding, injury to spermatic vessels or vas deferens, infection, fever) or late complications (failure to achieve satisfactory scrotal site or testicular atrophy), duration of surgery, LOS, the frequency of uROR, and the number of ReAd-s. In addition, testicular volume was calculated for each patient before surgery and at follow-up at 6 months using the following formula: testicular volume = 0.52 × length × width^2^ [19]. The obtained testicular volumes were compared to normal reference ranges within the age group derived from the published literature [20].

### 2.5. Surgical Technique

**Figure 2:**
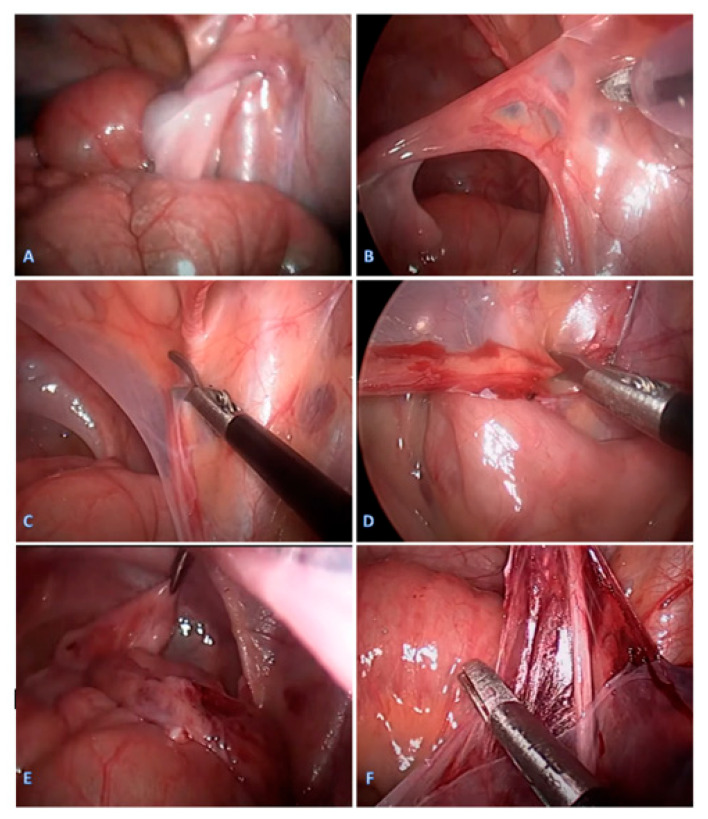
A single-stage, vessels preserving, laparoscopic orchiopexy in an 11-month-old pediatric patient with intra-abdominal testis: (**A**) Identification of the right testis near the iliac blood vessels; (**B**) pulling the testis with grasping forceps and identifying the spermatic cord vessels and vas deferens with blood vessels; (**C**) opening the peritoneum and separating the vas deferens from the posterior peritoneum; (**D**) carefully separating the spermatic cord vessels from the peritoneum; (**E**) inserting the grasping forceps medially to the epigastric blood vessels and pulling the testis into the scrotum; (**F**) additional dissection of the peritoneal bands.

In all the patients, a single-stage, vessel-preserving, laparoscopic approach was performed under general anesthesia, sparing the testicular blood vessels, with the patient in the supine position. The surgeon was on the opposite side of the undescended testis, the assistant was on the opposite side of the surgeon, and the operating nurse was at the side of the surgeon. A pneumoperitoneum of 6–8 mmHg, depending on the patient’s weight, was established using a Veress needle. The first 3.5 mm trocar was placed supraumbillicaly, and a 3.5 mm laparoscope (Karl Storz, Tuttlingen, Germany) was introduced. After inspection of the abdominal cavity, if a testis was identified (Figure 2A), two additional 3.5 mm trocars were inserted, one in the left or right lower abdomen lateral to the rectus muscle and another under the left or right costal arch. After identification of the testicular position, the testis was pulled with grasping forceps (Figure 2B). If presented, the gubernaculum was identified and resected with laparoscopic scissors after electrocautery was applied. Then, the testis was pulled, the posterior peritoneal wall was opened, and the vas deferens was separated from the posterior peritoneum (Figure 2C). After that, the spermatic cord vessels were carefully separated from the peritoneum, a scrotal incision was made, and a grasper was inserted through the inguinal canal into the abdominal cavity, medial to the epigastric blood vessels. Adequate spermatic cord length was considered in all cases when the testis should reach the opposite inguinal ring. The testis was grasped and gently pulled toward the scrotum (Figure 2E). If necessary, additional dissection of the peritoneal bands was performed until the testis was fixed in the middle of the scrotum without tension (Figure 2F). A second view into the abdominal cavity was performed to determine the correct position of the spermatic cord. At the end of the procedure, CO_2_ was removed from the peritoneal cavity, and the incisions were closed with braided adhesive skin tape (3M^TM^ Steri-Strip^TM^, Neuss, Germany).

### 2.6. Postoperative Protocol and Follow-Up

Oral nutrition was started two hours after surgery. Paracetamol (Perfalgan, Bristol-Myers Squibb S.r.l., Agen, France) was administered at a dose of 10–15 mg/kg for pain relief. All the patients were discharged within 24 h after surgery. The patients were followed up in our outpatient clinic. The adhesive skin tape was removed at the visit during the first week. The follow-up program included physical examination at one, six, and twelve months after surgery and then once a year to assess the presence of late complications (failure to achieve satisfactory scrotal site or testicular atrophy). At the six-month follow-up, an ultrasound examination of the testis was performed to measure the testicular volume.

### 2.7. Statistical Analysis

Statistical Package for the Social Sciences—SPSS 28.0 (IBM Corp, Armonk, NY, USA) and Microsoft Excel for Windows version 11.0 (Microsoft Corporation, Redmond, WA, USA) were used for the statistical analysis. The median and interquartile range (IQR) were used to describe the distribution of quantitative data, whereas the categorical data were described as absolute numbers and percentages. The Wilcoxon matched-pairs signed-rank test was used to compare the pre- and postoperative testicular volume values. All *p* values less than 0.05 were considered significant.

## 3. Results

### 3.1. Demographic Characteristics and Clinical Data of the Patients with Intra-Abdominal Cryptorchid Testes

A total of 51 laparoscopic explorations in children with impalpable undescended testes were performed. Of these, single-stage, vessels-preserving, laparoscopic orchiopexies was performed in 32 children (34 testes, two cases were bilateral). The median age at the time of surgery was 10 months (IQR 9, 13). The majority of the children (*n* = 24; 75%) were less than 12 months of age at the time of surgery. Associated anomalies were found in 7 (21.9%) children. The demographic characteristics and clinical data of the patients with intra-abdominal cryptorchid testes who underwent single-stage, vessels-preserving, laparoscopic orchiopexies are shown in Table 1.

### 3.2. Intraoperative Findings and Clinical Outcomes of the Intra-Abdominal Testes

Intraoperative findings and clinical outcomes were analyzed for 34 intra-abdominally located testes in which single-stage, vessels-preserving, laparoscopic orchiopexies were performed. A normal testis was found in 24 patients (70.6%), while in 10 cases (29.4%), hypotrophic testes were visualized. The majority of the testes were located near the internal ring (*n* = 19; 55.9%), while in the remaining cases, the testes were located near the iliac blood vessels. The median duration of the surgical procedure was 37.5 min (IQR 33, 42.5). The length of hospital stay was one day for all the children. No intraoperative complications were recorded. One child developed a wound infection at the site of the umbilical trocar and was managed conservatively. In 2 cases (5.5%), testicular atrophy was noted during the long-term follow-up. In three cases, during the follow-up period, the testis was found in a higher position in scrotum, but after 1–3 years, in two cases, the position was normal, while in one case, it remained at the same scrotal position. During the long-term follow-up, with a median of 35 months (IQR 19, 60.5), the total success rate was 94.5%. No cases of ReAd, uROR, or redo surgery were noted. The intraoperative findings and clinical outcomes of the children who underwent single-stage laparoscopic orchiopexies because of intra-abdominal testes are shown in Table 2.

### 3.3. Comparison of Testicular Volume before and Six Months after Surgery

The volume of each patient’s testicles was measured via ultrasound before and six months after the laparoscopic orchiopexy. The median testicular length prior to and after the laparoscopic procedure was 13.5 mm (IQR 12, 15) and 14 mm (IQR 11.5, 16), respectively (*p* = 0.313). The median testicular width prior to and after the laparoscopic procedure was 7 mm (IQR 7, 8) and 8 mm (IQR 7, 8), respectively (*p* = 0.158). Finally, the median of testicular volume at the 6-month follow-up increased from 0.31 mL (IQR 0.28, 0.43) to 0.40 mL (IQR 0.33, 0.53) (*p* = 0.017) (Table 3).

## 4. Discussion

The purpose of this single-institutional retrospective study was to prove the safety and long-term efficacy of single-stage, vessel-preserving, laparoscopic orchidopexies for intra-abdominal testicular retention in pediatric patients. Efficacy is defined as a postoperative intra-scrotal testis without atrophy. In our study, the previously mentioned technique was performed in cases of intra-abdominal testes located from the iliac blood vessels to the internal inguinal ring. After a long-term follow-up, the atrophy rate as well as the success rate was acceptable. Also, the intraoperative complications noted in our series were generally low. Therefore, our study unequivocally demonstrates that the single-stage, vessel-preserving, laparoscopic orchidopexies provide the expected outcome.

Since intra-abdominal testicular retention has been a relatively common diagnosis in male infants, many studies have been conducted with the aim of determining optimal treatment [21]. Recently, the standard of surgical care of intra-abdominal testicular retention has been a laparoscopic orchiopexy [11]. Whether it would be managed as a single-stage or staged procedure depends on the surgeons’ preferences as well as the position of the testes [22]. Compared with the open technique, laparoscopic orchiopexies have significant proven advantages and fewer complications [15,23,24,25]. The majority of surgeons consider the Fowler–Stephens (FS) procedure to be the treatment of choice for intra-abdominal testes in pediatric patients. Since the following method involves dissection of the spermatic blood vessels, Esposito et al. have shown that despite proper testicular descent, a low degree of vascularization is ultimately possible. According to them, this cannot be considered a successful outcome. Consequently, they recommended the vessel-preserving technique because it does not affect testicular circulation [23]. That might reduce the risk of testicular atrophy, and it is also considered to be a minimally invasive technique [15,23,25].

Several studies have described the outcomes of vessel-preserving laparoscopic orchiopexies as well as the outcomes of FS orchiopexies [13,26,27,28,29]. According to these studies, when considering both FS approaches, vessel-preserving laparoscopic orchiopexies provide a higher success rate. However, neither of them resulted in a recorded volume alteration after a follow-up period. Also, Powell et al. did not document the precise position of the retained testicle in the abdomen [13]. Due to the lack of standardized classification of the testicular position in the abdomen, some authors adopted their own specific definitions for the testicular position. Therefore, Samadi et al. adopted the following nomenclature. High-intra-abdominal testicles are those located more proximal to the iliac vessels, and low-intra-abdominal-testes are those located between the iliac vessels and the internal inguinal ring [26]. Baker et al. defined the testicular position based on their distance from the internal ring as <2 cm and >2 cm from the internal ring [27]. Moursy et al. identified the testicular position as low or high according to whether or not the testis could be pulled over to reach the contralateral internal inguinal ring [29].

In a review of 61 cases of intra-abdominal testes, Powell et al. investigated the surgical patterns of seven different surgeons. Each of them chose the technique they preferred. In their series, half of the cases underwent a vessel-preserving orchiopexy, while the remainder were treated either by a single-stage or two-stage FS orchiopexy. They reported testicular atrophy in 6.5% of the cases and successful outcomes in 96.7% of those who underwent the vessel-preserving laparoscopic orchiopexy. The atrophy rates for those who underwent single-staged and two-staged FS orchiopexies were 25% and 12.5%, while the success rates for the same groups were 75% and 87.5%, respectively [13].

Similarly, in a study of 101 cases of intra-abdominal testes by Chang et al., 80 children underwent laparoscopic surgeries. During the laparoscopies, they identified intraabdominal testes in 46 of the patients, iliac in 14, the internal ring in 22, ‘peeping’ in 12, behind the bladder in 3, and intra-canalicular in 4. Vessel-preserving laparoscopic orchiopexies was used for 72 testes. The authors did not record any testicular atrophy, and the success rate was 92%. The other 29 underwent either single-stage or two-stage FS orchiopexies. Their atrophy rates were 16% and 14%. Hence, the overall success of the FS procedures was 85%. They also implied that testicular atrophy might be more likely in cases of previous surgical procedures or dissection around the vas deferens [28].

Samadi at al. were in agreement with previous studies. At the time of the laparoscopy, 58% of the testicles were at the level of the iliac vessels or higher (high intra-abdominal), 22% were between the iliac vessels and the internal ring (low intra-abdominal), 16% were peeping, 3% were intracanalicular, and 1% were retro-vesical. A total of 203 procedures were performed in the 173 children. Vessel-preserving laparoscopic orchiopexies were performed in 70.5% of the children, while 29.5% of the children were treated with a single-stage or two-stage FS orchiopexy. In the first group, they also did not record a single case of testicular atrophy, and the success rate was 97%, while in the second group, the atrophy rate was 7% and the success rate was 90%. Ultimately, they did not specify which approach they used with regard to the testicular position [26].

It is also important to note that Baker et al. had similar outcomes. In their study of 310 cases of intra-abdominal testes, 252 children underwent laparoscopic surgeries. Atrophy was recorded in 2.2% of the cases, and successful outcomes were reported in 97.2% of the cases that underwent a vessel-preserving laparoscopic orchiopexy. This refers to 85.5% of testicles located <2 cm and 42.8% of testicles located >2 cm from the internal inguinal ring. The atrophy rates for those that underwent either a single-stage and two-stage FS orchiopexy were 22.2% and 10.3%, while the success rates for the same groups were 74.1% and 87.9%, respectively [27].

In an examination of 78 intra-abdominal testes by Moursy et al., 45 testes were identified as high (FS in 43; orchiectomy in 2 atrophic testes) and 33 as low (vessel-preserving laparoscopic orchiopexies). The overall success rate was 100% of those who underwent a vessel-preserving laparoscopic orchiopexy and 88.8% of those who underwent a FS orchiopexy [29].

Al Hindi et al. evaluated the outcomes of vessel-preserving laparoscopic orchiopexies in 19 patients. At laparoscopy, 10 testes were identified above the internal ring, 5 near iliac vessels, and 4 close to the kidney. During follow-up, there was not a single case of testicular atrophy, and the overall success rate was 89.5% [12]. Similar findings were noted in another study by Alzahem, who reported significantly better outcomes when vessel-preserving laparoscopic orchiopexies were performed. In their study, the overall success rates for vessel-preserving laparoscopic orchiopexies and FS orchioexies were 88% and 63%, respectively [30].

In our study, we also analyzed the testicular volume and showed the possibility of testicular growth after an orchiopexy. After a six-month follow-up, we noted statistically significant increases in testicular volume. Al Hindi et al., in addition to a high success rate, also showed significantly increased testicular volumes after orchiopexies. In their study, the testicular growth rate was higher than normal [12]. Therefore, changes in testicular volume during a follow-up period may be one of the success criteria [31,32,33,34,35].

There are a few limitations, including retrospective character of this study, the data used from a single center, and the relatively small sample size.

## 5. Conclusions

To conclude, our study indicates the utility of the single-stage, vessel-preserving laparoscopic orchiopexy. Therefore, this procedure can be considered to be the ‘gold standard’ for the treatment of the intra-abdominal testes that are located from the iliac vessels to the internal ring. Randomized controlled trials are needed to confirm the validity of this recommendation.

## Figures and Tables

**Table 1:** Demographic characteristics and clinical data of the patients (*n* = 32).

Parameter	Value
Demographic characteristics	
Age (months)	10 (9, 13)
<12 months	24 (75)
12–24 months	6 (18.8)
24–36 months	1 (3.1)
36–48 months	1 (3.1)
>48 months	0 (0)
Height (cm)	84.5 (81, 86)
Weight (kg)	10 (9, 12)
BMI (kg/m^2^)	14.9 (13.4, 15.6)
Clinical data of patients	
Lateralization	
Right	14 (43.8)
Left	16 (50)
Bilateral	2 (6.2)
Associated anomalies	7 (21.9)
Cardiac abnormalities	3 (9.4)
Hematological abnormalities	1 (3.1)
Metabolic abnormalities	1 (3.1)
Down syndrome	2 (6.3)

Data presented as median (IQR) or *n* (%); BMI—body mass index.

**Table 2:** Intraoperative findings and clinical outcomes of the intra-abdominal cryptorchid testes (*n* = 34).

Variables	Values
Intraoperative findings	
Normal testis	24 (70.6)
Hypotrophic testis	10 (29.4)
Atrophic testis	0 (0)
Intra-abdominal position of the testis	
Near the internal ring	19 (55.9)
Near the iliac blood vessels	15 (44.1)
Near the kidney	0 (0)
Duration of surgery (min)	37.5 (33, 42.5)
Length of hospital stay (days)	1 (1, 1)
Complications	
Wound infection	1 (2.8)
Testicular atrophy	2 (5.5)
Higher position in the scrotum	3 (8.3)
Follow-up (months)	35 (19, 60.5)
Reoperation	0 (0)
ReAd	0 (0)
uROR	0 (0)

Data presented as median (IQR) or *n* (%); ReAd—readmissions within 30 days of index surgery; uROR—unplanned return to the operating room.

**Table 3:** Comparison of testicular volume before and six months after surgery (*n* = 34).

	Before Surgery	Six Months after Surgery	*p* *
Testicular length (mm)	13.5 (12, 15)	14 (11.5, 16)	0.313
Testicular width (mm)	7 (7, 8)	8 (7, 8)	0.158
Testicular volume (mL)	0.31 (0.28, 0.43)	0.40 (0.33, 0.53)	0.017

Data presented as median (IQR); * Wilcoxon matched-pairs signed-rank test.

## Data Availability

The data supporting this study’s findings are available upon request from the corresponding author.
